# Maternal Diabetes Impairs Insulin and IGF-1 Receptor Expression and Signaling in Human Placenta

**DOI:** 10.3389/fendo.2021.621680

**Published:** 2021-03-10

**Authors:** Andrea Tumminia, Nunzio M. Scalisi, Agostino Milluzzo, Giuseppe Ettore, Riccardo Vigneri, Laura Sciacca

**Affiliations:** ^1^ Endocrinology, Department of Clinical and Experimental Medicine, University of Catania Medical School, Catania, Italy; ^2^ Obstetrics and Gynecology Unit, Azienda di Rilievo Nazionale e di Alta Specializzazione (ARNAS) Garibaldi, Catania, Italy; ^3^ Catania Section, Institute of Crystallography, National Research Council, CNR, Catania, Italy

**Keywords:** type 1 diabetes, gestational diabetes, adverse outcomes, HbA1c, placenta, insulin receptor, insulin-like growth factor-1 receptor

## Abstract

**Background:**

Maternal high blood glucose during pregnancy increases the risk for both maternal and fetal adverse outcomes. The mechanisms underlying the regulator effects of hyperglycemia on placental development and growth have not been fully illustrated yet. The placenta expresses high amounts of both insulin receptor (IR) and insulin-like growth factor receptor (IGF-1R). It has been reported that the placenta of diabetic women has structural and functional alterations and the insulin/IGF system is likely to play a role in these changes. The aim of the present study was to measure the content of IR and IGF-1R and their phosphorylation in the placenta of women with type 1 diabetes mellitus (T1D) or with gestational diabetes mellitus (GDM) compared to women with normal glucose tolerance (NGT) during pregnancy.

**Methods:**

Placental tissues were obtained from 80 Caucasian women with a singleton pregnancy. In particular, we collected placenta samples from 20 T1D patients, 20 GDM patients and 40 NGT women during pregnancy. Clinical characteristics and anthropometric measures of all women as well as delivery and newborn characteristics were recorded. Patients were also subdivided on the basis of peripartum glycemia either ≥90 mg/dl or <90 mg/dl, regardless of the diagnosis.

**Results:**

In T1D patients, a higher rate of adverse outcomes was observed. Compared to the GDM women, the T1D group showed significantly higher average capillary blood glucose levels at the third trimester of pregnancy and at peripartum, and higher third-trimester HbA1c values. In both T1D and GDM women, HbA1c values during pregnancy correlated with glucose values in the peripartum period (R-squared 0.14, p=0.02). A positive correlation was observed between phosphorylation of placental IR and the glucose levels during the third trimester of GDM and T1D pregnancy (R-squared 0.21, p=0.003). In the placenta of T1D patients, IGF-1R phosphorylation and IR isoform A (IR-A) expression were significantly increased (p=0.006 and p=0.040, respectively), compared to the NGT women. Moreover, IGF-1R phosphorylation was significantly increased (p<0.0001) in the placenta of patients with peripartum glucose >90 mg/dl, while IR-A expression was increased in those with peripartum blood glucose higher than 120 mg/dl (p=0.046).

**Conclusions:**

To the best of our knowledge, our study represents the first one in which an increased maternal blood glucose level during pregnancy is associated with an increased IGF-1R phosphorylation and IR-A expression in the placenta. Both these mechanisms can promote an excessive fetal growth.

## Introduction

In women with pre-gestational diabetes a good glycemic control before and during pregnancy is essential to reduce the risk of adverse outcomes. Maternal high blood glucose during pregnancy increases the risk of both maternal and fetal/neonatal adverse outcomes. Maternal glucose can cross the placenta (1–5) which, in turn, can send signals into the maternal and fetal circulations. The precise nature of these signals and their regulation in response to glucose changes are still unknown (6). In particular, the mechanisms underlying the regulator effects of hyperglycemia on placental development and growth have not been fully investigated yet. It has been reported that the placenta of diabetic women is less efficient since it presents structural and functional alterations (7) and that the insulin/IGF system is likely to play a role in these changes (8, 9). The placenta expresses high amounts of both IR and IGF-1R, as well as IGF-1 and IGF-2, which appear to be involved in most mechanisms of placental development and function. In pregnant women with T1D or GDM, the insulin/IGF system is, of course, dysregulated and these alterations may influence the release of placental nutrients to the fetus (8, 10, 11). In type 1 diabetic women, a positive correlation between the umbilical cord IGFBP3 and IGF-1 levels and macrosomia (12, 13), and also between maternal glycosylated hemoglobin (HbA1c) and umbilical cord IGF-1 and IGF-2 levels, has been found; this latter parameter, in turn, correlates with birth weight (14). An impaired vascular function has been reported in the placenta of diabetic mothers, and this might lead to deleterious consequences for the fetal development and growth as well as for the health of the mother. Placental hypervascularization in diabetic women is mainly attributed to an increased angiogenesis and insulin might play an important modulatory role in human placental endothelial cells. Fetal hyperinsulinemia consequent to maternal hyperglycemia can stimulate endothelial cell proliferation, probably through the IR-A (15). The altered expression of insulin receptor isoforms A and B influences the reduced fetoplacental vascular dilatation in response to insulin observed in GDM (16). In early pregnancy, the insulin receptors are mainly expressed in the syncytiotrophoblast, facing the maternal circulation, while at term, they are mainly expressed in the placental endothelial cells, facing the fetal circulation (17). These findings suggest a different role and effects of insulin in the first trimester, mainly due to the maternal plasma insulin levels, or in term pregnancy, mainly due to fetal plasma levels. Decreased fetal insulin levels result in reduced placental weight, supporting the hypothesis that fetal insulin is a growth factor for the placenta. The use of insulin analogs has improved the possibility of reaching a good metabolic control, but the effects of insulin or its analogs in the structure/function of the placenta (i.e. altered expression of growth factors, altered angiogenesis, etc.), and the consequent effects on fetal development are poorly known (18–21).

Few evidence are available on the post-receptor insulin signaling in the placenta. In animal models, the maternal hyperglycemic environment leads to a decreased ERK and AKT phosphorylation during the placenta development, and consequently, to abnormalities of embryonal developmental (22, 23). It is not clear whether the timing of onset of hyperglycemia, at the beginning (as in T1D) or at the end of pregnancy (as in GDM), differently affects placental development and growth.

The present study aimed to evaluate both the content and activation of IR and IGF-1R in the placenta of T1D patients (having pre-pregnancy hyperglycemia) compared to GDM patients (having hyperglycemia initiated during pregnancy) and to women with normal glucose tolerance during pregnancy. We also evaluated the expression and activation of IR and IGF-1R in the placenta in relationship to the maternal blood glucose during labor and delivery.

## Methods

### Clinical Study

Placental tissues were obtained from 80 Caucasian women with a singleton pregnancy, selected among patients followed at the Garibaldi-Nesima Medical Center in Catania (Italy), who delivered in the same Hospital and agreed to participate by informed consent.

The studied women included three groups (**Table 1**):

- 20 T1D women followed at the Diabetic Center since the pre-conception period (planned pregnancy, n=4) or early gestation (unplanned pregnancy, n=16). These patients were regularly visited by a specialized team (physician, dietitian and nurse) every 1 or 2 weeks according to our established protocol. Glycemic control was evaluated by checking patient’s self-monitoring of blood glucose during each visit and by measuring HbA1c every 1–2 months, depending on the team judgement. Patients with severe overweight (BMI > 27 kg/m^2^), or not compliant with the visit protocol or with advanced micro- or macro-vascular chronic diabetes complications, were excluded.
- 20 GDM patients, diagnosed according to the criteria of the International Association of Diabetes and Pregnancy Study Group (24, 25), and followed, since the GDM diagnosis at the second (n=3) or third (n=17) trimester of pregnancy, according to the same protocol of T1D patients. Women with severe overweight (BMI > 27 kg/m^2^) or not compliant with the visit protocol were excluded.
- 40 NGT women during pregnancy, in a 2:1 ratio with the other two groups. Women with severe overweight (BMI > 27 kg/m^2^) were excluded. The clinical characteristics of NGT pregnant women were extracted from the medical records of the Obstetric Center of our Medical Center.

**Table 1:** Clinical and anthropometrical characteristics of the studied population.

	T1D(n = 20)	GDM(n = 20)	NGT(n = 40)	P
Age (years)	27 ± 6*	34 ± 5	32 ± 6	<0.01
Pre-pregnancy BMI (kg/m^2^)	24 ± 3	24 ± 3	21 ± 3**	<0.01
Weight gain at end of pregnancy (kg)	14 ± 5	13 ± 4	12 ± 4***	<0.05
HbA1c (%)				
Peri-conception	7.9 ± 1.5			
First trimester	7.3 ± 1.0			
Second trimester	6.6 ± 0.7			
Third trimester	6.5 ± 0.7	5.4 ± 0.3		<0.01
Blood glucose (mg/dl)				
At 28 weeks	129 ± 26	110 ± 13		<0.05
Third trimester	119 ± 16	108 ± 14		<0.05
Peripartum	123 ± 47	87 ± 14		<0.01
Systolic blood pressure (mmHg)	108 ± 8	110 ± 12	115 ± 9	0.54	
Diastolic blood pressure (mmHg)	66 ± 7	68 ± 6	70 ± 8	0.32	

Data are presented as mean ± standard deviation (SD).

T1D, type 1 diabetes; GDM, gestational diabetes mellitus; NGT, normal glucose tolerance; HbA1c, glycated hemoglobin; BMI, body mass index.

*Indicates statistical significance value T1D vs. GDM and vs. NGT.

**Indicates statistical significance value NGT vs. GDM and vs. T1D.

***Indicates statistical significance value NGT vs. T1D.

The number of the cases was estimated on the basis of the T1D pregnant patients referred to our diabetes center yearly and followed up simultaneously in the gynecology and obstetrics center in the same hospital, in a placenta collection period of about 3 years.

The following data were recorded for each studied subject (**Table 1**): age, pre-pregnancy BMI, weight gain during gestation, systolic and diastolic blood pressure. Moreover, for T1D and GDM patients, data on glycemic control and for T1D onset or progression of diabetes-related micro-vascular complications were obtained by using the albumin/creatinine ratio for nephropathy and ophthalmoscopy for retinopathy (26).

Glycemic control was assessed by capillary blood glucose and HbA1c values. Although the dosage of HbA1c cannot be considered clinically useful in GDM pregnancy (except for the initial assessment of the degree of glycemic control), a study conducted on T1D patients has demonstrated a predictive value of HbA1c on the occurrence of fetal macrosomia or LGA, neonatal hypoglycemia and the time of delivery, even at an advanced stage of gestation (27).

For all cases, the delivery and newborn characteristics were recorded, including neonatal gestational age, length and weight, APGAR scores at 1 and 5 min, Rohrer ponderal index (birth weight g x 100/height in cm^3^) and blood glucose levels. Placenta weight was also measured (**Table 2**).

**Table 2:** Pregnancy and neonatal outcomes.

	T1D (n = 20)	GDM (n = 20)	NGT (n = 40)	P
Gestational age at delivery (weeks)	37 ± 2*	38 ± 1	39 ± 1	<0.05
Preterm birth (%)	25*	5	2.5	<0.01
Cesarean section (%)	70*	45	35	<0.05
Weight at birth (g)	3451 ± 820*	3269 ± 527	3270 ± 359	<0.01
Macrosomia (>4000 g) (%) Large for gestational age (%)	25* 25	10 0	2.5 0	<0.01 NA
Ponderal index (g/cm^3^ x 100)	2.82 ± 0.5*	2.55 ± 0.2	2.62 ± 0.2	<0.05
Neonatal hypoglycemia (%)	15	5	2.5	0.08
Weight of the placenta (g)	549 ± 103	593 ± 162	562 ± 91	0.70
APGAR score after 1 min	8.3 ± 0.9	8.5 ± 1.0	8.9 ± 0.6	0.44
APGAR score after 5 min	9.5 ± 0.8	9.6 ± 0.6	9.8 ± 0.5	0.76

Data are presented as mean ± standard deviation (SD) or percentages (%).

Births occurring before 37 weeks of gestation are considered as preterm.

T1D, type 1 diabetes; GDM, gestational diabetes mellitus; NGT, normal glucose tolerance; NA, not applicable.

*Indicates statistical significance value in T1D vs. GDM and vs. NGT.

All procedures performed in this study were conducted in accordance with the ethical standards of the Declaration of Helsinki and its later amendments.

### *In Vitro* Studies

#### Placental Tissue Preparation

Immediately after delivery, placentas were collected and weighted, and tissue specimens were excised from the central region of the fetal side, deprived of the amniotic membrane.

Samples were rapidly cut into small pieces (1 cm^3^) and immediately frozen at -80°C. The frozen placental tissue was powdered by using the Mikro Dismembrator U (B. Braun—Biotech International GmbH, Schwarzenberger Weg 73.79, D-34212 Melsungen, Germany) in liquid N_2_.

#### Insulin and IGF-1 Receptor Measurements and Phosphorylation

Powdered placental tissue was homogenized for 1 min in ice-cold lysis buffer containing 50 mM HEPES (pH 7.4), 150 mM NaCl, 1% Nonidet P-40, 10 mM sodium fluoride, 10% glycerol, 1 mM MgCl2, 1 mM CaCl2, 2 mm phenylmethylsulfonylfluoride, 2 mm sodium orthovanadate, 5 μg/ml leupeptin, 5.9 μg/ml Aprotinin, 1x Complete Roche—Protease Inhibitor Cocktail Tablets. The tissue homogenate was incubated for 60 min at 4°C in continuous rotation and then centrifuged at 14,000 g for 60 min. The resulting supernatant was collected and assayed for protein concentration using the Bradford dye-binding assay kit with BSA as a standard.

For immunoprecipitation studies, equal amounts of placental extracts (1 mg) were subjected to immunoprecipitation between protein G-Sepharose beads and anti-insulin receptor NB400-142 (MA-20) from Novus Biologicals (Littleton, USA) or anti-IGF-1R (Ab-1) mouse mAb (αIR3) from Calbiochem (San Diego, CA) for 2 h at 4°C, washed three times with lysis buffer, then eluted with Laemmli buffer 2 x for 5 min at 100°C.

For immunoblotting studies, equal amounts of solubilized placental proteins were resolved by electrophoresis on Criterion TGX gels 4%–15% (Bio-Rad, USA), directly or after immunoprecipitation with the specific antibodies, as indicated above. The resolved proteins were transferred to nitrocellulose blotting membrane (Amersham Protran GE Healthcare Life Sciences, GE) using a transfer buffer containing 192 mM glycine, 25 mM Tris pH 8,3, 20% (vol/vol) methanol. To reduce non-specific binding, the membranes were incubated in TBST supplemented with 5% nonfat dry milk for 1 h at room temperature and then incubated for 1 h at room temperature with the appropriated antibodies.

Polyclonal anti-IGF-1 receptor beta-subunit was purchased from Cell Signaling Technology, Inc. (Beverly, MA). Polyclonal anti-insulin receptor beta-subunit was purchased from Santa Cruz Biotechnology, Inc. (Santa Cruz, CA).

The proteins were visualized by enhanced chemiluminescence using ECL™ anti-rabbit or anti-mouse IgG horseradish peroxidase linked whole antibody (from sheep) (GE Healthcare, UK) and quantified by densitometric analysis using ImageJ analysis software (1.42 q, Wayne Rasband, National Institutes of Health, USA).

#### Insulin Receptor Isoforms’ Transcript Measurement

RNA was extracted by Trizol RNA isolation (Thermo Fisher Scientific, Waltham, Massachusetts, USA) through the gentle MACS TM Dissociator instrument using the M-Tubes (Miltenyi Biotec, Germany). Two μg RNA were reverse transcribed into cDNA using High Capacity cDNA Reverse Transcription Kits (Applied Biosystems, Foster City, California, USA). Synthesized cDNA was then combined in PCR reaction using forward and reverse primers to detect A and B isoform of the insulin receptor as previously described (28). The signal intensity was quantified by densitometric analysis using ImageJ analysis software.

#### Statistical Analysis

Statistical analysis was performed using the one-way analysis of variance (ANOVA). Fisher exact test or χ2 test were used when appropriate for categorical variables. Linear and logistic regressions were used for the correlation between variables (with continuous or dichotomous outcome, respectively) and the Student’s t test for comparison between the two groups. Quantitative data are expressed as mean ± standard deviation (SD), qualitative data as percentage (%). A value of p<0.05 was considered statistically significant.

Newman-Keuls Multiple Comparison Test was performed when comparison among groups by One-way non-parametric ANOVA showed significant differences.

Data analyses were performed using the StatView and SPSS statistical package software (version 15; SPSS Inc., Chicago, IL, USA).

## Results

### Clinical Characteristics, Glucose Control, and Pregnancy Outcomes

Clinical and anthropometrical features of the studied cohort are summarized in **Table 1**. Both age and pre-pregnancy BMI were significantly different in the three groups and, therefore, the statistical analysis was corrected for these two parameters.

In T1D women the peri-conception HbA1c was 7.9% ± 1.5. Seventeen (85%) T1D patients were on multi-injective insulin therapy, while three of them (15%) were on insulin pump therapy. Only four T1D patients (20%) planned the pregnancy, reaching peri-conception HbA1c <6.5%. Women with GDM were treated with either diet alone (n=9, 45%) or insulin in addition to diet (55%).

Compared to the GDM women, T1D women showed significantly higher average capillary blood glucose levels at the third trimester of pregnancy (108 ± 14 mg/dl *vs*. 119 ± 16 mg/dl, p<0.05) and at peripartum (87 ± 14 mg/dl *vs*. 123 ± 47 mg/dl, p<0.01). T1D patients also had higher third-trimester HbA1c values (6.5% ± 0.7 *vs*. 5.4% ± 0.3, p<0.01) (**Table 1**).

In both T1D and GDM women HbA1c values during pregnancy correlated with glucose values in the peripartum period (R-squared 0.14, p=0.02) suggesting that peripartum values reflected the level of metabolic control during pregnancy.

The mean weight gain during pregnancy was higher in T1D patients (14.3 ± 5.4 kg) compared to both GDM and NGT women (**Table 1**) while systolic and diastolic blood pressure values were similar in the three groups (**Table 1**).

Gestational age at delivery was significantly lower (p<0.05) in the T1D group (37 ± 2 weeks), compared to the GDM group (38 ± 1 weeks) and to the NGT group (39 ± 1 weeks). T1D patients had a higher rate of preterm birth (25%, p<0.01) (**Table 2**). In T1D group, also the percentage of Caesarean section was higher (70%) compared to the other two groups (45% and 35% for the GDM and the NGT women, respectively) (p<0.05).

In T1D patients, a higher rate of neonatal hypoglycemia (15%) was observed as well as less favorable neonatal anthropometric indices: higher percentage of macrosomia (25%), weight at birth (3451 ± 820 g) and Rohrer ponderal index (2.82 ± 0.5 g/cm^3^), compared to GDM and NGT women. On the contrary, the Apgar score was similar in the three groups (**Table 2**).

In T1D and GDM women, HbA1c values during pregnancy were correlated with macrosomia/LGA (OR 3.33, 95% CI 1.36–8.15, p= 0.008).

Other diabetes-related complications during pregnancy were rare: one T1D woman developed microalbuminuria and two developed non-proliferative retinopathy. Preeclampsia occurred in one patient with GDM.

### Placental Insulin and IGF-1 Receptor Content and Phosphorylation

**Figure 1:**
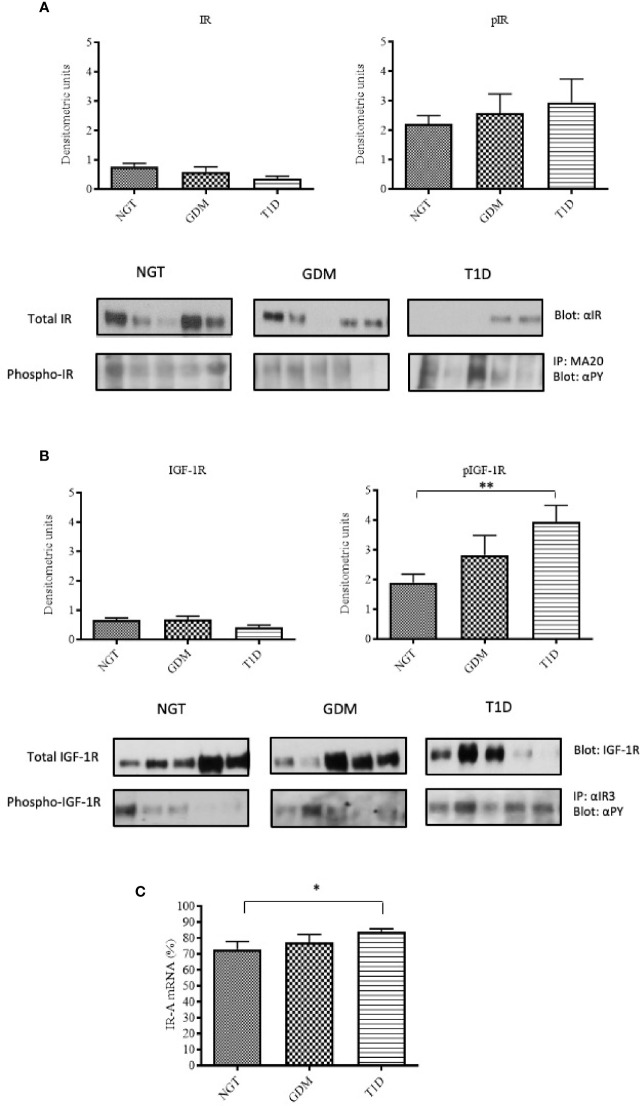
IR and IGF-1R content and phosphorylation in placental tissues. Insulin receptor protein content was detected by immunoblotting with a polyclonal anti-insulin receptor β-subunit. **(A)** The protein content of IGF-1R in placenta samples was detected by immunoblotting using a polyclonal anti-IGF-1R β-subunit. **(B)** Solubilized placental tissues were subjected to immunoprecipitation with an anti-IR or anti IGF-1R. The resulting immune complexes were resolved by SDS-PAGE and immunoblotting with an anti-PY antibody to detect receptor phosphorylation **(A, B)**. Protein content was normalized against β-actin, whereas phosphorylated proteins were normalized against their total amount. Each bar represents mean ± SE of all placental samples (n=40 for NGT, n=20 for GDM and n=20 for T1D). A representative Western-blot is included in the bottom (five different patients for each group). IR-A mRNA expression in placental tissue measured by RT-PCR. The bar graphs show the quantitation of IR-A mRNA (mean ± SE) in all samples for each group **(C)**. *p < 0.05; **p < 0.001, after adjusting, in multivariate logistic regression models, for age, pre-pregnancy BMI, gestational age at delivery and type of delivery.

**Figure 2:**
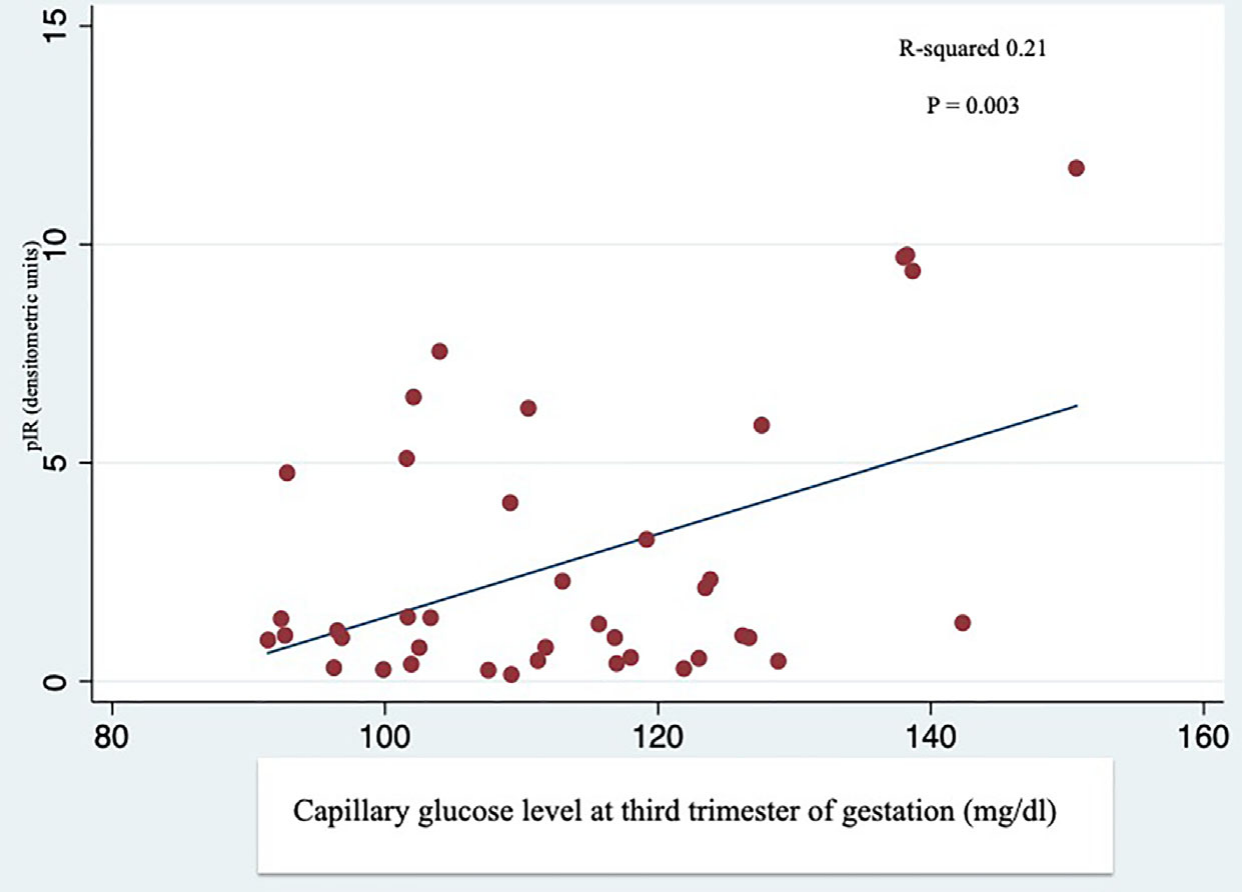
Linear regression analysis between phosphorylation of IR (pIR) and average capillary glucose levels during the third trimester. Self-monitoring of blood glucose performed by GDM and T1D patients during the third trimester of gestation was correlated with the IR phosphorylation in the placenta of GDM and T1D women.

The total IR protein content was reduced in the placenta of T1D women (-53% compared to the NGT group and -40% compared to the GDM group) (**Figure 1A**). In contrast, in T1D group, IR phosphorylation was slightly increased (+33% and +13% compared to the NGT and GDM groups, respectively) (**Figure 1A**). The IR content and phosphorylation were similar in GDM women treated only with diet (45% of GDM cases) and in GDM women treated also with insulin. A positive and statistically significant correlation was observed at the linear regression analysis between phosphorylation of placental IR and the glucose levels during the third trimester of GDM and T1D pregnancy (R-squared 0.21, p=0.003) (**Figure 2**).

The total IGF-1R content was also slightly reduced in the T1D patients compared to the other two groups, but, in contrast to the IR, IGF-1R phosphorylation was strongly increased (p=0.006) compared to the NGT group (**Figure 1B**).

We found that, if IR-A was the prevalent IR isoform in the placenta of all three groups (NGT 73%, GDM 77%, T1D 83%), it was significantly increased in the placenta of T1D patients compared to the NGT group (p=0.040) (**Figure 1C**).

### Maternal Blood Glucose Level, Placental Insulin and IGF-1 Receptors Content and Activation

**Figure 3:**
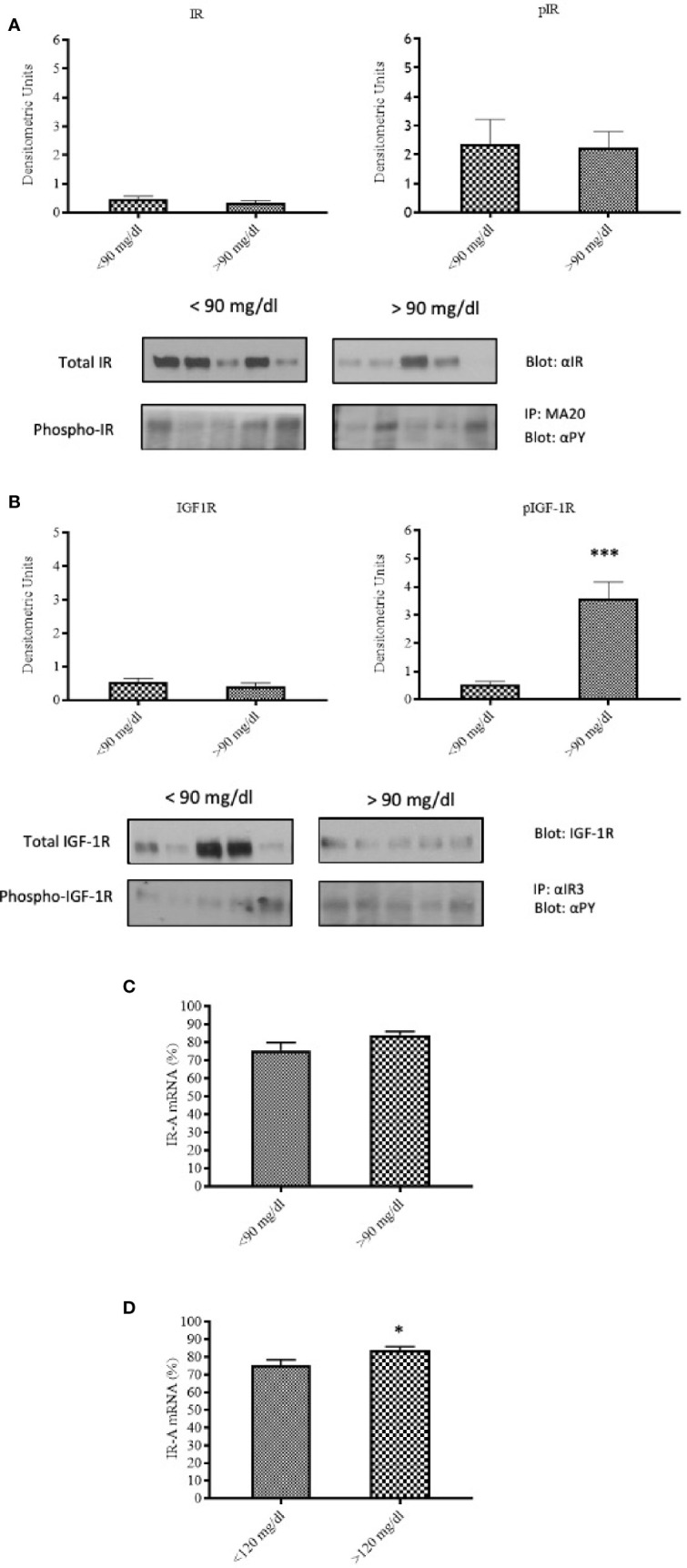
IR and IGF-1R content and activation in relationship to the maternal capillary blood glucose level during labor and delivery. IR content and phosphorylation **(A)**, and IGF-1R content and phosphorylation **(B)** in placental tissues of patients with peripartum blood glucose <90 mg/dl (N=25) or ≥90 mg/dl (N=15). Each bar represents mean ± SE of the samples indicated above. ***p < 0.0001. A representative Western-blot is included in the bottom (five different patients for each group). IR-A mRNA expression in placental tissues of patients with peripartum average blood glucose <90 mg/dl (N=25) or ≥90 mg/dl (N=15) **(C)** and of patients with peri-partum average blood glucose <120 mg/dl (N=30) or ≥120 mg/dl (N=10) **(D)**. Each bar represents mean ± SE of the samples indicated above. *p < 0.05.

To evaluate the influence of maternal glucose level at peripartum on the IR and IGF-1R expression and phosphorylation, and IR-A/IR-B expression ratio in the placenta, patients were subdivided on the basis of peripartum (within 6 h of delivery) glycemia either ≥90 mg/dl or <90 mg/dl, regardless of the diagnosis. IR content and phosphorylation were similar in placental tissues from patients with peripartum glucose level either ≥90 mg/dl or <90 mg/dl (**Figure 3A**). In contrast to IR, IGF-1R phosphorylation was significantly increased (p<0.0001) in patients with peripartum glucose >90 mg/dl (**Figure 3B**).

IR-A expression was slightly, non-significantly increased in the placenta of patients with peripartum glucose level ≥90 mg/dl (**Figure 3C**). The difference became statistically significant (p=0.046) when a cut-off level of 120 mg/dl was used instead of 90 (**Figure 3D**).

Therefore, peripartum hyperglycemia, which may reflect worse metabolic control during pregnancy, is associated to increased IGF-1R phosphorylation and increased expression of the IR-A isoform.

## Discussion

Hyperglycemia has detrimental effects on the placenta development and growth (7) with consequent increased risk for adverse maternal and/or fetal outcomes. In presence of high blood glucose, the expression and signaling of different growth factors can be altered. In a rabbit model, an exposure to maternal hyperglycemia in the periconception period is able to irreversibly affect the feto-placental phenotype, through a structural and molecular alteration of placenta (29). IR and IGF-1R and their ligands have a relevant role in the placental and fetal growth but their expression and activity in placenta of diabetic women is poorly understood.

Our study provides some novel information on the field. By comparing pregnant women with T1D and GDM, we were able to observe the effects of a pre-existing hyperglycemia (T1D) from hyperglycemia occurring in the second half of pregnancy (GDM), or pregnancy without hyperglycemia. Of course, both T1D and GDM pregnant women received appropriate treatment to achieve a good glycemic control (30), and this treatment may have influenced the expression and phosphorylation of IR and IGF-1R in the placenta at term, thus obscuring the differences. However, some differences remained, and probably contributed to the worse outcomes of T1D pregnant women. Comparing the glycemic control in T1D and GDM starting from the 28^th^ week of gestation (when GDM was diagnosed in most of patients), both glycemia and HbA1c values were higher in T1D patients than in GDM patients, despite in T1D patients HbA1c was significantly lower in the third trimester compared to the conception level (6.5 ± 0.7% *vs*. 7.8 ± 1.4%, p <0.001). In T1D patients, the significantly higher glycemic level was associated with a higher percentage of macrosomia or LGA, as well as the newborn weight and the ponderal index. These latter indexes are positively correlated with the placenta weight, which in turn positively correlates with HbA1c values. These results strongly suggest a close link between maternal glycemic control and the fetal-placental unit growth.

Regarding the expression of receptors, both IR and IGF-1R content was reduced in the placenta of T1D patients but IGF-1R was significantly more phosphorylated in T1D placenta and this activation was significantly increased in patients with peripartum glucose level >90 mg/dl. These results are consistent with the existing data that demonstrated a phosphorylation of IGF-1R increased by two to three times in pregnant rats with streptozocin-induced insulinopenic diabetes (31), compared to the control group. IGF-1R is known to play an important role on the control of placental and fetal growth. Fetal IGF-1 gene deletion causes a reduction in placental weight (32), and the same holds for fetal weight when an IGF-1R mutation is present (33). Moreover, intrauterine growth retardation (IUGR) is associated with a reduction of placental IGF-1R (34), while IGF-1R gene amplification (three copies) causes an increased weight and length of the newborns (35). It has been reported that the insulin receptor is less expressed in the placenta of GDM women treated with diet alone than in the placenta of GDM women treated also with insulin (10). We did not find such a difference probably because of the similar pre-pregnancy BMI in the two groups (23.8 ± 2.7 kg/m^2^ in diet alone group *vs*. 24.3 ± 3.6 kg/m^2^ in insulin group, p=0.75) as well as the similar insulin resistance index (HOMA-IR) (1.70 ± 0.81 in diet alone group *vs*. 1.96 ± 1.49 in insulin group, p=0.67). However, our different results might also depend on the sample size of these subgroups, not sufficient to highlight differences in insulin receptor expression between GDM women treated with only diet and those treated also with insulin.

The importance of the expression and function of the insulin receptor family in the placenta is confirmed by the IR isoform biology. The IR mediates both metabolic and mitogenic effects, but IR-A and IGF-1R are both receptors for IGF-2, a well-known growth factor during pregnancy (36). IR-A is more expressed in proliferating cells (37), and IR-A expression was increased in the placenta of T1D women and in those with peripartum blood glucose higher than 120 mg/dl. These data suggest that higher level of blood glucose can regulate the expression of IR-A, modulating fetal growth. Moreover, we observed a significant correlation between placental IR phosphorylation and third trimester blood glucose level, as well as a positive correlation between HbA1c during pregnancy and glucose levels in the peripartum period. High level of HbA1c is a strong risk factor for macrosomia/LGA (OR 3.33). Therefore, these findings confirm that a strict glycemic control is necessary from conception to delivery in order to reduce adverse outcomes. Our results also indicate that the expression and activation of placental receptors are possible biological mechanisms contributing to the hyperglycemia-related complications during pregnancy. Despite the great difficulties in patients with T1D, the achievement of an ambitious goal of glycemic control is required and to this aim the new technologies (insulin pump therapy, continuous glucose monitoring) can be helpful (38–40).

In conclusion, to the best of our knowledge, our study is the first demonstrating that higher HbA1c values are associated with fetal macrosomia/LGA and are related to higher blood glucose level in the peripartum period, suggesting the increased phosphorylation of IGF-1R and the increased expression of IR-A among its possible mechanisms. Both these mechanisms can promote an excessive fetal growth. Further studies are necessary to better understand the role of IR and IGF-1R in physiological pregnancies, particularly those complicated by diabetes.

## Data Availability Statement

The raw data supporting the conclusions of this article will be made available upon reasonable request directed to LS. lsciacca@unict.it

## Ethics Statement

Ethical review and approval were not required for the study on human participants in accordance with the local legislation and institutional requirements. The patients/participants provided their written informed consent to participate in this study.

## Author Contributions

AT contributed to the acquisition, analysis, and interpretation of data, wrote part of the manuscript, drafted tables and some figures, and reviewed the paper. NMS performed *in vitro* experiments, contributed to the acquisition, analysis, and interpretation of data, wrote part of the manuscript, drafted some figures, and revised the paper. AM contributed to the acquisition, analysis, and interpretation of data, and reviewed the manuscript. GE contributed to data acquisition. RV contributed to data interpretation and reviewed the manuscript. LS contributed to the study design, data interpretation, wrote and reviewed the manuscript. All authors contributed to the article and approved the submitted version.

## Conflict of Interest

The authors declare that the research was conducted in the absence of any commercial or financial relationships that could be construed as a potential conflict of interest.
